# Learning engagement; nursing students' experiences in an online environment at a university

**DOI:** 10.1002/nop2.1564

**Published:** 2022-12-24

**Authors:** Takaedza Munangatire, Lea Indjamba

**Affiliations:** ^1^ Department of Nursing University of Namibia Rundu Namibia

**Keywords:** engagement, experiences, learning, nursing, students

## Abstract

**Aim:**

The aim of this study was to understand students' engagement with learning in an online environment at a university in Namibia.

**Design:**

A qualitative descriptive design was applied.

**Methods:**

Data were collected using in‐depth telephonic interviews among 12 purposively selected nursing students. Data were analysed thematically.

**Results:**

While the students did practice engagement, their understanding of learning engagement was not clear. The students considered online learning to be a safe space for engagement that enhanced active participation. They also noted that it forced them to reflect on their pre‐existing knowledge before learning new information and ultimately enhanced their understanding of the learning material. The findings of this research suggest that online platforms and apps can support nursing students' learning engagement, in particular behavioural engagement, but they are less conducive to cognitive and affective engagement.

## 1 INTRODUCTION

Engagement refers to active learning, where students and lecturers interact with, participate in and contribute to, the teaching and learning process (Hudson, 2015). Kuh (2009) provided a two‐way definition of engagement, which includes the time and effort that students commit to learning activities and what higher education institutions do to promote students' participation in teaching and learning activities. These activities include class attendance, self‐study, practical work and interactions with the lecturer or other students to attain the required outcomes (Groccia, 2018). Students' engagement can also be described as the depth of students' learning, the extent of attention paid and self‐directedness (Deschaine & Whale, 2017; Fisher et al., 2021).

Engagement goes beyond mere participation in teaching and learning activities; there are also emotions involved and psychological aspects (Chan et al., 2021). The three essential types of engagement are thus behavioural, emotional and cognitive engagement (Bowden et al., 2021). Behavioural engagement integrates a student's individual style and approach to learning, their drive to study and their strategies to succeed (Herrmann, 2013), while emotional engagement explains the level of affective investment a student puts into their learning and their emotional obligation to complete the learning course (Brown et al., 2017). Cognitive engagement focuses on the psychological aspects that are applied to learning and meeting the required learning outcomes (Hudson, 2015).

## 2 BACKGROUND

### 2.1 Online learning and students' engagement

Online learning or e‐learning refers to a form of teaching and learning that is conducted through the Internet (Chan et al., 2021). It has been described as the use of digital technology to facilitate learning through forums where real‐time interactions are possible, or asynchronous learning through the use of tools such as pre‐recorded videos (Siah et al., 2022). In online learning, digital tools such as mobile devices or computers hosting web‐based apps or educational software provide a platform for learning to take place (Haslam, 2021; Palvia et al., 2018). Software such as Moodle, Blackboard, Microsoft Teams and Zoom, among others, have different learning capabilities that support online learning. With the advent of the COVID‐19 pandemic, it was predicted that online learning would become predominant (Kim et al., 2021).

Even before being forced into online learning, academics had already been investigating how online education can be used to enhance learning engagement (Chan et al., 2021). Before adopting online learning, students' learning experiences, including engagement, should be considered (Bond et al., 2020), that is online learning must be interesting (Dixson, 2015). Unfortunately, this has not been the case. The rise in online learning has rather posed a risk to learners' engagement, and the dropout rate of students may grow if online learning is not implemented in a better, more engaging, manner (Choy & Quek, 2016; Wan et al., 2020).

In any form, learning online or face‐to‐face students' engagement is important and is an essential determinant of success in higher education (Dixson, 2015; Fisher et al., 2021; Hwang & Shin, 2018; Moonaghi et al., 2015). Learning engagement can improve students' retention and satisfaction (Choo et al., 2020), while preparing them better for the world of work (Hwang & Shin, 2018). With good engagement, students have a better chance of learning effectively and attaining the desired learning outcomes (Gonzalez et al., 2020; Natarajan & Joseph, 2022). There is evidence to suggest that learning engagement is not always attained despite its importance in nursing education (Ghasemi et al., 2020). Therefore, there is a need to continually explore ways of engaging students, particularly in online learning environments (Chan et al., 2021).

With outbreak of COVID‐19 pandemic, teaching and learning abruptly shifted to online. The online learning was introduced without adequate preparation and due consideration for its suitability for different contexts and has been referred to as emergency remote teaching (Bond et al., 2021). Under these circumstances, the key challenge faced by educational institutions was how to engage students in an online learning environment. Evidence regarding students' experiences of engagement with online learning is still lacking, yet such knowledge can inform future students' engagement practices of online learning.

### 2.2 Literature review

Previous research has shown that in the field of nursing, a blended learning approach that incorporates online learning improves students' engagement and subsequently their academic performance, in both practical and theoretical aspects (Busebaia & John, 2020; Tang et al., 2017). These findings do not reveal the extent to which online learning promotes engagement, however. In addition, a separate study on online learning using video conferencing found that this method is less engaging than the face‐to‐face lecture method (Ramos‐Morcillo et al., 2020). According to same study, communication between students and lecturers was limited during online teaching; however, the recording option was considered to be a positive aspect as it allowed for flexibility in terms of revisiting a lecture.

In contrast, a recent study conducted during the pandemic found that the use of self‐paced video lectures, in combination with instant online activities and postclass activities, was an effective strategy for engaging learners (Busebaia & John, 2020). In addition, the use of YouTube was reported to significantly engage students in learning activities and improve the quality of the learning experience, as students could watch the videos at their own time and pace, giving them a chance to reflect on the content (Johnston et al., 2018). Furthermore, the innovative use of a flipped classroom strategy and instant quizzes got students actively engaged cognitively in a fully online course (Chan et al., 2020). Given the many other innovations that were implemented during online learning in the pandemic, further research is needed to explore the concept of students' engagement in online learning.

According to Carley (2015), when used effectively, online technology can promote active learning and students' engagement in the learning process. Web‐based technologies such as Kahoot and Poll Everywhere were found to increase students' participation during lectures (Bond et al., 2020; Fotaris et al., 2016). Furthermore, Broussard and Wilson (2018) suggested that the use of web‐based courses could enhance students' engagement, without specifying how these courses get students engaged. One way in which web‐based technology could increase engagement is through asynchronous discussions, where students can actively participate and engage with the lecturer and other students (Dixson, 2015). On the contrary, a recent study suggested that although asynchronous discussions helped students remain connected with the lecturer and other students, the students considered the discussions to be substandard (Ramos‐Morcillo et al., 2020). These findings reflect the sentiments of Rodrigues and Zealand (2016), who echoed that technology use alone, without relevant techniques such as reflections, problem‐based learning and gaming, will not result in students' engagement.

This study aimed to explore undergraduate nursing students' experiences with online learning engagement. The students were enrolled in a four‐year nursing degree programme on a full‐time basis and were all taking courses such as nursing science, community nursing and midwifery. All the theoretical aspects of the degree were online, while practical training took place face‐to‐face. The online learning was delivered through Moodle for asynchronous sessions, Zoom, Google meet and Microsoft teams for synchronous sessions. YouTube and recorded videos were shared through links in Moodle and WhatsApp groups. WhatsApp was also used for communication and small group real‐time discussions. In this study, the research question was: What were the nursing students' experiences of engagement in an online learning environment?

## 3 METHODS

### 3.1 Aim

The aim of the study was to describe the learning engagement of nursing students in an online learning environment.

### 3.2 Design

The concept of engagement in online learning in nursing education is relatively understudied, with the constructs that measure engagement also remaining a subject needing further enquiry. Resultantly, a qualitative descriptive design was used to describe the nursing students' experiences of learning engagement in an online learning environment because the variable experience is best measured through qualitative data (*Sandelowski*, 2010).

### 3.3 Sampling

The study participants were recruited from a national university in Namibia which has satellite campuses across the country. The university offers programmes in various fields, with total enrolment of over 25,000 students. Nursing programmes are offered at four of the satellite campuses. The inclusion criteria were as follows: (a) All second‐ to fourth‐year students, as they had experienced online learning at the time of study, and (b) students in the undergraduate nursing programme at any of the four university campuses. Both convenience and purposive sampling were applied to select the participants (Moser & Korstjens, 2017). An invitation to participate in the study was sent out to students via WhatsApp groups, and interested students were encouraged to contact the researchers directly on a voluntary basis. Those who expressed an interest in participating were screened to ensure that only those students who had the most exposure to all online tools participated in the study (not all students were able to fully participate in online classes due to challenges with Internet connectivity, availability and electronic devices to connect for online classes). A total of 12 participants took part in the study, with four students from each of the four university campuses.

### 3.4 Procedures

Before the COVID‐19 pandemic, face‐to‐face lecturing was the dominant teaching strategy for the theoretical components of the nursing course at the university's Department of Nursing Science. In March 2020, a COVID‐19 pandemic protocol was implemented in Namibia, which moved all theoretical teaching and learning online. Face‐to‐face lecturing was thus replaced by online learning facilitated through Moodle, Google Meet, Zoom, Panopto and WhatsApp, among others.

### 3.5 Data collection

Twelve semi‐structured individual interviews were conducted with the participants between September and December 2020. The researchers facilitated the interviews telephonically using the English language. The researchers requested that the participants find a quiet and comfortable space in which to answer the interview questions, which were audio recorded using a voice recorder. Each interview took 15–30 min. Telephonic interviews on speakerphone were necessary in order to comply with the COVID‐19 pandemic regulations and to fulfil the ethical principles of beneficence by not putting the participants at risk. Using a speakerphone allowed for a better‐quality recording. During the participant recruitment process and subsequent interviews, pseudonyms (numbers) were used to identify the participants to ensure their anonymity. During the interviews, the participants were asked about their understanding of engagement in teaching and learning; how they went about engaging with learning online; what technological tools helped them to be engaged; and in what ways they were engaged with learning. This was accompanied by probing questions that led them to add more details and explanations on their experiences. The semi‐structured interview guide in this study was developed by the authors based on the literature on engagement. Input from experts was used to enhance the validity of the interview guide. Furthermore, the first interview was used as a pilot test, but the data were used in the study because they were judged to be valid. All the recordings and the data transcripts were stored on the password‐protected personal computer of one of the researchers and were deleted from the voice recorder.

### 3.6 Data analysis

The researchers transcribed the interviews verbatim, before analysing the data thematically to identify the themes underpinning students' engagement with the help of ATLAS.ti version 9 software. Braun and Clarke's (2006) thematic analysis approach was used, which took the following steps. Initially, the researchers read each interview transcript several times to familiarize themselves with the data. The transcripts were then read to search for data units to code, which were related to the students' experiences with learning engagement. These codes were scrutinized to identify and create categories and subsequently themes. This process involved both researchers, and the final themes were a result of the researchers' interpretation of the data based on the participants' words.

### 3.7 Trustworthiness

The use of two analysts improved credibility, as they were able to critique each other's interpretations of the data. Furthermore, when there were differences in interpreting a transcript, a discussion was held not to reach a consensus, but to provide a deep discourse and a broader interpretation of the data. Such a measure is referred to as crystallization, which Varpio et al. (2017) recommended as an expanded way of ensuring credibility.

Data saturation was not used to determine the sample size, as data saturation was not practically possible to attain. Saturation requires that interview saturation and coding saturation are reached, which is nearly impossible to attain. As an analysis was done concurrently with the data collection, after 10 interviews the data were found sufficient to answer the research questions. This was established by coding each interview transcript several times and comparing the codes. Code sufficiency was judged to have been reached when researchers could not generate any new or total unrelated codes from the transcripts. Regarding meaning sufficiency, the authors analysed the transcripts and codes repeatedly at different points and sufficiency was judged to have been attained when no new significant meanings were emerging. Two extra interviews were conducted to further enhance the richness of the data, however, and to confirm that data sufficiency had been reached. In addition, member checking was done to get a reflective perspective on the findings from the participants, rather than checking the correctness of the researchers' interpretations. The researchers believe that qualitative data analysis is a constructionist process and interpretation will differ, hence there is no need to push for agreement, but rather to get a variety of interpretations to ensure a rich meaning. Varpio et al. (2021) argued that data are not merely the voices of participants, but a reflection of their voices as shaped and interpreted by researchers.

## 4 FINDINGS

The 12 participants' ages ranged from 20 to 24 years old. Half of the participants were female, and the other half were male. Exactly half of the participants were fourth‐year nursing students (6), followed by 25% third‐year students (3) and 25% second‐year students (3).

### 4.1 Theme 1: Understanding of engagement

This theme describes how the students understood the concept of engagement during a lecture. According to the interviewees, engagement refers to participation during the teaching and learning process. The students' understanding of engagement reflected that they were used to the lecture method. The word “lecture” was used constantly in explaining engagement. Several ways of engagement were highlighted, including active listening, note taking, questioning and contributing to the course content.

Engagement itself is getting involved in something, so I think students' engagement in a lecture is students participating in lessons; getting involved. [P1]

Besides actively listening during lectures, the participants explained that students' engagement could also be students following along with their lecturers during the presentation and listening attentively.

What I understand by students' engagement in a lecture is just when the student is following in class when the lecture is teaching; that's by listening attentively. [P12]

The students further explained that engagement during lectures also involves taking notes when the lecturer is presenting and writing notes on points that may need clarification.

Writing notes [and] asking questions where the student doesn't understand. [P3]

In addition, the students thought that giving opinions about what is being presented and adding additional information to what has already been said is also a form of engagement. Asking questions and being asked questions by the lecturer or other students was further considered to be engagement.

They are giving in their inputs; like maybe they are giving in their topics. [P7]

That's the involvements of students during lectures that involve asking questions and answering. [P4]

The students' understanding of engagement was limited to what happens during lectures or presentations, with more emphasis on behavioural and partly cognitive engagement, and less on affective engagement.

### 4.2 Theme 2: Online learning as a safe space for engagement

In this theme, the students experienced the online environment to be a safe space for learning engagement. They described how their engagement was negatively affected in face‐to‐face learning, and highlighted how the online platform helped overcome the challenges presented by face‐to‐face teaching. The participants also indicated that in classes there is limited chance to engage with learning because when there are many students, the lecturer may fail to control them.

In my experience what I think has limited my engagement is that in lectures there are some students that talk too much and don't give chances to others to talk. [P10]

However, through online platforms such as WhatsApp, Google Meet and Moodle, a safe platform was created where students could contribute to the learning process without fear of having to speak face‐to‐face. Short messages in apps such as Google Meet, and the ability to anonymize names in WhatsApp, enabled students to contribute freely.

We have been using Moodle, WhatsApp also; it has been a good experience though. It's really improved students' participation because, and there are actually those ones that are scared in face‐to‐face in giving answers or being wrong or being that so when it's an online thing like WhatsApp and Moodle makes it easy. [P4]

### 4.3 Theme 3: Enhanced questioning and participation

This theme is related to how technology enhances students' learning engagement. The participants thought that short message apps such as WhatsApp prompt learning engagement through instant questions and responses. The participants highlighted that interactions are easy and fast, as all students have the chance to participate in solving a problem. In addition, the students experienced team work in problem‐solving.

…for me I think WhatsApp is good for interactions; a lecturer poses a question or discussion or topic many people can come in at the same time and interact and help each other and solve a problem… it's also very simple to use, you just type almost like an SMS and you send and it's very easy. [P11]

The use of Zoom and Google Meet made the students feel like they were physically present with the lecturers during presentations. The students thought this helped them to gain confidence and participate during the classes as they could type their answers or questions in the chat options provided.

We join the live discussion virtually but it's like reality platform as if it was in real face‐to‐face classes and students engage [and] ask questions. Information is clarified immediately at that time… also one can use instant messaging to interact with lecturer and others in real time, helping me to be confident enough to participate. [P9]

Furthermore, being able to use Google and other search engines during a presentation allowed the students to search for additional information or verify what was being presented, helping them to understand the topic under discussion better and hence contribute more to the presentation.

Search engines that we were talking about helps to break down certain things that you never had a clue of it, breaks it down to an understanding, and even provides us with clues to help us know what they are talking about. [P10]

### 4.4 Theme 4: Enhanced activation of prior knowledge and knowledge retention

This theme describes how the students think technology could activate prior knowledge and help knowledge retention post a presentation. Asynchronous platforms such as Moodle provide students with course content before an online presentation, preparing the students for engagement. Gaining this knowledge on a topic before a discussion or presentation helps them to connect with the lecturer and other students and contribute during the presentation or discussion.

What I basically do is go through content on Moodle relating to that topic, so when the topic is discussed or when we talk about the topic I already know what the lecture is referring to, and if I found something that I didn't understand I could quickly point it out to the lecturer during the presentation or discussion. [P5]

In addition, students reported using YouTube to watch videos on specific topics before and after the lecture. This allowed them to gain more knowledge on what was going to be taught or something that had already been taught, helping them to engage more with the course content and engage with others in the presentation or discussion.

[When] I go for a lecture or discussion online or WhatsApp I would use my own knowledge, like the knowledge I gained from the internet from watching YouTube videos and other sources so like, ‘Yay, that gave me enough ideas enough knowledge to contribute to that topic’. [P8]

These practices reported by students mirror a flipped classroom, but this time there was no face‐to‐face engagement as it was replaced with online engagement. The participants highlighted that having prior knowledge on a topic enabled them to be more active in presentations and online discussions.

## 5 DISCUSSION

The research found that nursing students had an acceptable understanding of the concept of engagement. Their understanding demonstrated that they are aware of the behavioural and cognitive aspects of learning engagement, but less aware of affective engagement. They demonstrated good knowledge of the activities that constitute engagement, and although they did not indicate some activities when asked, they did highlight them as they were explaining more about how they are engaged. While this study sought to directly ascertain students' understanding of engagement, literature has focused more objectively on defining engagement (Hudson, 2015), describing how students learn to engage (Brown et al., 2017), and demonstrating engagement (Johnston et al., 2018). It is thus important that students' conceptions of engagement be explored further, as this will guide how they go about learning and demonstrating engagement. It can be said that one cannot develop learning engagement skills before one understands what it means to engage. A claim can thus be put forward that the road to students' learning engagement starts with understanding the concept of engagement.

The study revealed that the participants believed that online learning is a safe learning platform, which encourages students to participate, in contrast to face‐to‐face teaching. These findings complement the argument by Raiman et al. (2017) that online learning is more engaging than face‐to‐face learning. Nevertheless, some evidence still suggests that online learning alone is not as engaging as blended learning, hence more still needs to be done before online learning can be considered adequately engaging for students (Lawn et al., 2017). The students were of the opinion that instant messaging apps and options in WhatsApp, Moodle, Zoom and Google Meet make it easy for students to be engaged. This is in line with the literature, where web‐based technologies such as Kahoot and Poll Everywhere were shown to improve students' participation in the learning process (Fotaris et al., 2016). The study further clarified that instant messaging apps remove the boundaries of fear, increase the number of students who can participate, and increase the confidence of students to participate. This adds to the findings of previous studies that revealed that the use of web‐based apps enhances students' engagement, although these lacked details of how they actually get students engaged (Broussard & Wilson, 2018). This study confirms that online learning provides more tools and access for students to engage, but this only demonstrates behavioural engagement. Cognitive engagement, which involves the real learning and transformation that should happen in the students, received little attention. There is thus a risk of increasing the quantity of students' participation in learning activities, without a corresponding improvement in quality of students' learning.

In this study, the standards of facilitation and the use of online platforms in learning were not mentioned by students, which gave the impression that the participants generally had opportunities to engage with them. Existing evidence does show that it is only through the correct use and application of relevant techniques of reflection, problem‐based learning, lecturer innovation and support that students can be engaged with online learning (Ramos‐Morcillo et al., 2020; Rodrigues & Zealand, 2016). The findings of this study suggested that online learning can improve students' behavioural engagement, but cognitive engagement requires more than just a change in platform from face‐to‐face to online learning. The skills of reflection should therefore be developed among students to promote learning, regardless of the mode of learning. Subsequently, educators should deliberately teach and support students to develop reflection skills that are necessary for cognitive engagement in an online learning platform.

Online learning platforms make it easy to access course content pre‐ and postpresentations and discussions. The students thought that platforms such as Moodle, Panopto, YouTube and Zoom, which had recorded videos, activated prior knowledge and helped in knowledge retention post a presentation. This made students more engaged in discussions and with the content. These findings are in line with those of Johnston et al. (2018), who demonstrated that YouTube significantly engages students in learning activities and improves the quality of the learning experience through self‐paced and flexible learning. Making content available prior to a discussion or presentation creates an online version of a flipped classroom, where there is no face‐to‐face learning. Such an online‐only version of a flipped classroom got students engage cognitively in a full online course (Chan et al., 2020). However, the findings of this study did not concur with those of Ramos‐Morcillo et al. (2020), who claimed that video conferencing is a downgraded face‐to‐face lecture method with weak interactions between students and lecturers. The differences in this study's findings and that literature could be attributed to the use of video conferencing apps such as Zoom, where instant messages can be shared during recording, and the use of YouTube channels, where students can interact through the comments sections. Consequently, students in this study found the videos engaging, mainly due to the way they were used in online learning. This raises the question whether engagement is about the mode of learning, or about how the mode of learning is managed by the lecturer. For this reason, the role of educators is paramount when it comes to enhancing nursing students' engagement in online learning (Choy & Quek, 2016). There is need to adequately prepare educators on online pedagogies that can enhance nursing students' engagement.

Reflecting on the findings of this study, it can be seen that students' learning engagement in an online learning environment is not substantially different from students' learning engagement in a face‐to‐face environment. In both modes of learning, students need to understand engagement. Online engagement does provide a safe environment, tools and access for students to engage much more than the online environment, however. In an online environment, students may participate more, which may define some aspects of behavioural engagement such accommodating students' individual learning styles and studying strategies such as listening, watching videos and participating. There is nothing to suggest, however, that students in an online learning platform are driven to study more than those in a face‐to‐face class. Neither are they going to engage more cognitively or emotionally compared to those in the face‐to‐face mode. Based on this study, it can be said that nursing students need self‐directed learning skills to enhance their engagement when on online platforms. For successful engagement, nursing students can thus be educated on self‐directed learning to be prepared for online learning.

## 6 LIMITATIONS

This study was conducted at one university in Namibia, so it may not be a true reflection of the experiences of all nursing students in Namibia. The participants in this study used many platforms for online learning, and this could have impacted on their experiences of online learning. The findings could have been different if the participants all used one common mode or platform of online learning. Despite this, the findings provide a relevant snapshot on how online learning can impact students' learning engagement. Future studies should focus on large‐scale quantitative studies to measure students' learning engagement in online learning. Further studies are also needed to measure students' knowledge of learning engagement, rather than there being an assumption that students know what it means to engage. The students' lack of understanding about what engagement is could have compromised the data that were collected, if they were only focused on what they understood to be engagement.

## 7 CONCLUSION

The findings of this research suggest that online platforms and apps can support nursing students' learning engagement, in particular behavioural engagement, but they are less conducive to cognitive and affective engagement. Considering the paucity of data on students' engagement in online learning, there is a need for further innovations and studies to measure students' engagement in online learning, with a view to maximize learning engagement in online nursing education.

## AUTHOR CONTRIBUTIONS

LI and TM conceived and designed the study. LI collected and both LI and TM analysed the data.LI drafted the manuscript while TM made critical revisions on the paper and also supervised the study. All authors have agreed on the final version and meet at least one of the following criteria [recommended by the ICMJE (https://www.icmje.org/recommendations/)]:

- substantial contributions to conception and design, acquisition of data or analysis and interpretation of data;
- drafting the article or revising it critically for important intellectual content.

## FUNDING INFORMATION

No funding was given for this study.

## CONFLICT OF INTEREST

The authors declare that there is no conflict of interest.

## ETHICAL APPROVAL

The study was conducted according to the Helsinki Declaration and was held in accordance with the COVID‐19 pandemic protocols of Namibia and the World Health Organization (WHO). Ethical clearance and permission to conduct the study was obtained from the School of Nursing's Ethical Clearance Committee of the University (Ref: SoNE16/2020). Informed consent – both written (for students at the campus of the data collector) and verbal – was obtained from all the participants. Due to the COVID‐19 pandemic, the ethical committee approved verbal consent provided it was confirmed by the data recordings of the interviews. During recruitment, verbal and written explanations on the study were shared with the potential participants. The participants were made aware that they were free to decide whether to participate or not and that they could withdraw from the study at any time without any negative consequences. At the start of each interview, confirmation of the participant's consent was sought to ensure that they were still willing to take part in the study. While it was not possible to maintain anonymity in all situations (the data collector was a student at one of the campuses), the names and other personal information of the study participants were only exposed to the data collector.

## Data Availability

Data is available at a reasonable request from the authors.
